# Development and evaluation of new mask protocols for gene expression profiling in humans and chimpanzees

**DOI:** 10.1186/1471-2105-10-77

**Published:** 2009-03-05

**Authors:** Donna M Toleno, Gabriel Renaud, Tyra G Wolfsberg, Munirul Islam, Derek E Wildman, Kimberly D Siegmund, Joseph G Hacia

**Affiliations:** 1 Department of Biochemistry and Molecular Biology, University of Southern California, Los Angeles, CA 90089, USA; 2 National Human Genome Research Institute, National Institutes of Health, Bethesda, MD, 20892, USA; 3 Center for Molecular Medicine and Genetics, Wayne State University School of Medicine, Detroit, MI 48201, USA; 4 Department of Preventive Medicine, University of Southern California, Los Angeles, CA 90089, USA

## Abstract

**Background:**

Cross-species gene expression analyses using oligonucleotide microarrays designed to evaluate a single species can provide spurious results due to mismatches between the interrogated transcriptome and arrayed probes. Based on the most recent human and chimpanzee genome assemblies, we developed updated and accessible probe masking methods that allow human Affymetrix oligonucleotide microarrays to be used for robust genome-wide expression analyses in both species. In this process, only data from oligonucleotide probes predicted to have robust hybridization sensitivity and specificity for both transcriptomes are retained for analysis.

**Results:**

To characterize the utility of this resource, we applied our mask protocols to existing expression data from brains, livers, hearts, testes, and kidneys derived from both species and determined the effects probe numbers have on expression scores of specific transcripts. In all five tissues, probe sets with decreasing numbers of probes showed non-linear trends towards increased variation in expression scores. The relationships between expression variation and probe number in brain data closely matched those observed in simulated expression data sets subjected to random probe masking. However, there is evidence that additional factors affect the observed relationships between gene expression scores and probe number in tissues such as liver and kidney. In parallel, we observed that decreasing the number of probes within probe sets lead to linear increases in both gained and lost inferences of differential cross-species expression in all five tissues, which will affect the interpretation of expression data subject to masking.

**Conclusion:**

We introduce a readily implemented and updated resource for human and chimpanzee transcriptome analysis through a commonly used microarray platform. Based on empirical observations derived from the analysis of five distinct data sets, we provide novel guidelines for the interpretation of masked data that take the number of probes present in a given probe set into consideration. These guidelines are applicable to other customized applications that involve masking data from specific subsets of probes.

## Background

The development of gene expression microarray technology over a decade ago has revolutionized the analysis of the transcriptomes from numerous organisms. The earliest gene expression microarrays focused on widely-used experimental organisms, such as *Arabidopsis thaliana *[1], *Mus musculus *[2], *Saccharomyces cerevisiae *[3], *Drosophila melanogaster *[4], and *Caenorhabditis elegans *[5], in addition to humans [6]. In the intervening years, the number of commercially available species-specific whole genome expression microarrays has dramatically increased. Nevertheless, there are numerous species, such as African great apes (bonobos, chimpanzees, and gorillas), for which whole genome expression microarrays are not commercially available.

In such cases, gene expression is often conducted using microarrays designed to evaluate a closely-related species or organism (reviewed in ref. [7]). Several groups have employed commercially available human oligonucleotide microarrays comprised of multiple 25 mer probes to obtain gene expression profiles from African great ape tissues and cultured cells [8-14]. However, similar to observations from cross-species resequencing analyses [15,16], this comes at a price of underestimating the abundance of orthologous transcripts with poor affinity for the arrayed probes due to mismatches, as discussed in references [17-19].

One approach to address this problem is to remove (mask) data from probes predicted to have poor affinity for orthologous transcripts based on sequence information (reviewed in ref. [7]). This has been made possible by the development and use of algorithms that can map short oligonucleotide probe sequences to entire genomes and other sequence databases (e.g. methods described in references [20-30]). Several different strategies exist that range from masking all probes not perfectly matched to a given transcriptome [8,13,31] to masking only those probes with unfavorable hybridization properties based on predicted thermodynamic properties [32]. While multiple groups have examined the relationship between the number of probes within a probe set and the properties of resultant gene expression scores (e.g. references [27,33,34]), its effect on the comparative analysis of human and chimpanzee cross-species gene expression data sets has not been discussed in detail.

Here, we developed updated mask protocols for the analysis of human and chimpanzee gene profiles with commonly used Affymetrix human oligonucleotide microarrays. We first describe the development of new mask files which only retain data from probes that are perfectly matched to a single human and single chimpanzee genomic sequence. Next, we apply these masks to an existing publicly available oligonucleotide microarray gene expression data set representing five tissues derived from six humans and five chimpanzees [13]. We quantify the effects that altering the number of probes measuring the abundance of a given transcript have on intra- and interspecies gene expression comparisons. Based on our observations, we suggest general rules for the interpretation of gene expression scores using masking protocols.

## Results

### Properties of individual probes

**Figure 1:**
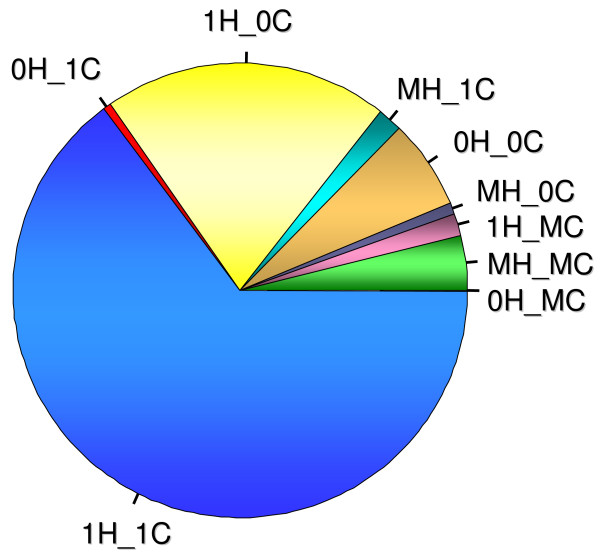
**Classification of the 604,258 probes within the Affymetrix U133Plus2 microarray**. The pie chart depicts the relative percentage of probes comprising each of the nine probe categories described in the Methods section.

We developed an algorithm to rapidly map short sequence tags to complete genomes (Renaud and Wolfsberg, unpublished) and used it to determine how many times each probe in the Human Genome U133Plus2 microarray (Affymetrix) had an exact match in the human and chimpanzee genomes. The bulk of the probes (86%) in the U133Plus2 microarray have exactly one match in the human genome (Table 1, Fig. 1). This is in contrast to 67% of the probes matching one time in the chimpanzee genome. Overall, 64.7% of individual probes showed one match in both the human and chimpanzee genomes.

**Table 1:** Classification of probes in the Affymetrix U133Plus2 microarray

		Human genome			
		No matches	Multiple matches	One match	Total
Chimpanzee Genome	No match	38,659 (6.40%)	4,745 (0.79%)	122,682 (20.30%)	166,086 (27.49%)
	
	Multiple matches	214 (0.04%)	23,350 (3.86%)	9,827 (1.63%)	33,391 (5.53%)
	
	One match	3,246 (0.54%)	10,568 (1.75%)	390,967 (64.70%)	404,781 (66.99%)
	
	Total	42,119 (6.97%)	38,663 (6.40%)	523,476 (86.63%)	604,258 (100%)

Human Genome Build 36.1 and Chimpanzee Genome Build 2.1 were used in these analyses.

We assigned probes into nine categories based upon the number of matches (0, 1, or Multiple) with the human (H) and chimpanzee (C) genomes (Methods). These included: 0H_0C, 0H_1C, 0H_MC, 1H_0C, 1H_1C, 1H_MC, MH_0C, MH_1C, and MH_MC. The 1H_1C and MH_MC categories are among the easiest to justify. The former involves conserved single gene sequences in both species while the latter at least in part reflects multi-copy gene families in humans and chimpanzees. The 1H_0C category reflects consequences of fixed sequence differences between the human and chimpanzee genomes. Below, we will discuss possible reasons for the remaining six probe categories, with the understanding that sequence errors or polymorphisms could influence each case.

Affymetrix currently employs a system in which the annotation of each probe set is classified into one of five categories based on the evidence available for the probe set interrogating the intended gene of interest. The five categories are designated A, B, C, E, and R, with A indicating the most direct evidence for a probe/transcript relationship. Of the 358 probe sets for which 11 probes are 0H_0C (no match to the chimpanzee or human genome), 182 (~51%) were provided an A level annotation. In contrast, ~81% of the 4,648 probe sets for which 11 probes are 1H_1C (match once in both species) were provided an A-level annotation. In addition to these sequence quality issues, probes designed to overlap splice junctions absent in human genomic sequences will also fall into the 0H_0C category.

Possible explanations for the 1H_MC, MH_1C, and MH_0C probe categories include assembly errors of multi-copy genomic regions, duplication or loss of genetic material in either lineage, or mutations in duplicated segments. The classification of probes in the two remaining categories (0H_MC and 0H_1C) was unexpected since these require one or more matches in the chimpanzee and none in the human genome. This could arise due to the positioning of probe sequences across splice junctions found in human cDNA sequences. Such probes would not match the human genome; however, they could match processed pseudogene sequences present in the chimpanzee genome.

Since our downstream cross-species (i.e. human versus chimpanzee) gene expression analyses would focus on data derived from the 1H_1C probes, we next evaluated the percentage of 1H_1C probes that were located in orthologous regions of the chimpanzee and human genomes. We determined if regions are orthologous by using the liftOver tool provided by the UCSC Genome Bioinformatics Group . We started with the coordinates of sequences that had a single hit in the human genome (1H), and used the liftOver tool to map them to the chimpanzee genome. We then compared the liftOver coordinates to the coordinates that we had obtained by aligning the sequences to the chimp genome. If a liftOver coordinate was within 100-nt of our coordinate, we counted the chimp hit as occurring in an orthologous region. Of the 390,967 sequences that have a single hit to both genomes (1H_1C), 388,044 (99.3%) hit orthologous regions in the chimpanzee and human genomes.

We then explored in further detail the 2923 1H_1C sequences that did not map to orthologous regions by liftOver. A total of 2488 of the 2923 sequences are either in an intron or an exon of a human gene, or within 5-kb upstream or downstream. Likewise, 2,021 of the 2,488 sequences were also in an intron or an exon of a chimpanzee gene, or within 5-kb upstream or downstream. Of these 2,021 genes, 1,660 are predicted to be human/chimpanzee orthologs.

Taking this additional information into consideration, we conclude that 389,704 (99.7%) of the 1H_1C probes map to orthologous regions in the chimpanzee and human genomes. This is especially impressive since the chimpanzee genome assembly used is of lower quality than the human, which would result in some probes being falsely identified as not mapping to orthologous regions in both genomes. Overall, these observations strongly support the use of 1H_1C probes for the analysis of human and chimpanzee gene expression profiles.

### General properties of probe sets

A total of 91.4% of probe sets (49,957 total) in the Human Genome U133Plus2 microarray had at least one probe removed in the initial masking process (i.e. contained at least one probe not in the 1H_1C category). In addition, 3,674 probe sets were completely eliminated from the most basic masking analysis (mask0, Table 2). These included 48 probe sets in the AFFX control category, which by design are not expected to match the human or chimpanzee genomes.

**Table 2:** Classification of probe sets based on number of 1H_1C probes

Filter	Minimum # of 1H_1C probes in a probe set	# of probe sets remaining
Mask0	1	51,001
Mask1	2	49,956
Mask2	3	48,876
Mask3	4	47,506
Mask4	5	45,402
Mask5	6	42,093
Mask6	7	37,103
Mask7	8	29,907
Mask8	9	21,192
Mask9	10	12,125
Mask10	11	4,967

Note that 54,675 probe sets are present in the U133Plus2 microarray.

**Figure 2:**
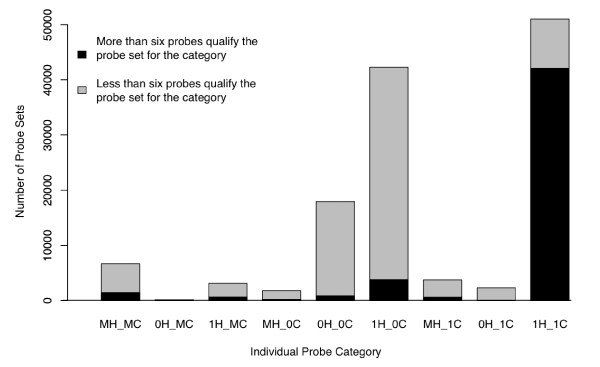
**Classification of the 54,675 probe sets within the Affymetrix U133Plus2 microarray**. The composition of probe sets with respect to probe categories is depicted. The height of each bar represents the number of probe sets (Y-axis) that contain a least one probe in the indicated category (X-axis). The gray segment of each bar represents the number of probe sets where less than six probes of the indicated category are present. The black segment comprising each bar represents the number of probe sets where more than six probes of the indicated category are present.

Next, we considered how many of the nine probe categories were represented in a given probe set. For each specified category, we determined the number of probe sets containing six or more probes (Fig. 2). These are designated as being 'category-enriched' probe sets. Interestingly, we noted deficits in the number of annotated genes in certain category-enriched probe sets. At the time of analysis, 37% of the 54,675 probe sets were annotated with a unique NCBI Entrez GeneID. A total of 2,371 (51%) of the 4,648 1H_1C_11 probe sets were annotated with unique Entrez Gene IDs. However, only 70 (8.1%) of the 0H_0C category-enriched probe sets (N = 862) were annotated. Likewise, only 289 (19.7%) of the MH_MC category-enriched probe sets (N = 1,464) were annotated. Strikingly, no Entrez GeneID was provided for any of the 0H_1C category-enriched probe sets (N = 35).

### Effect of probe number on estimates of intra- and interspecies expression variation

**Figure 3:**
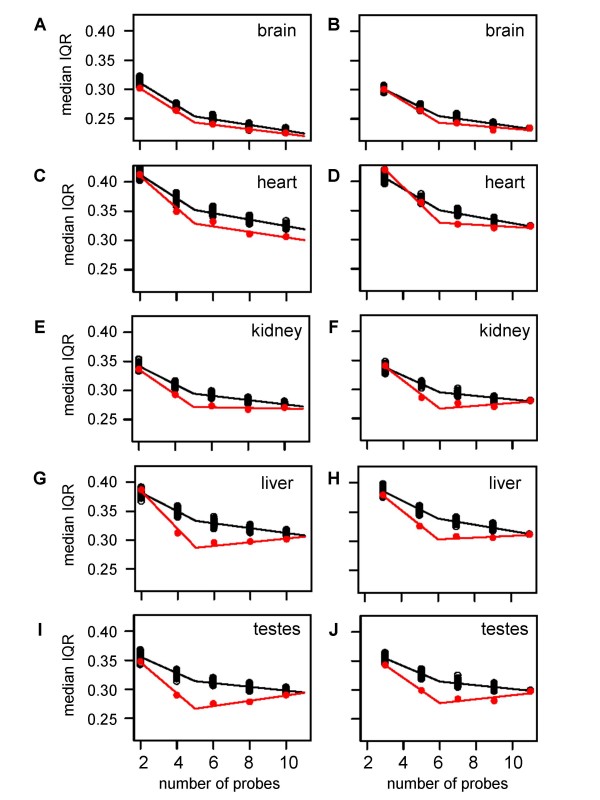
**Effects of probe number on the variation of gene expression scores**. Median interquartile ranges (IQRs) of expression scores (Y-axes) for the indicated tissue from all humans and chimpanzees are plotted in red relative to the number of 1H_1C probes remaining in a probe set (X-axes). Median IQRs are plotted in black for simulated data wherein different numbers of probes were randomly sampled from 1H_1C_11 probe sets (see text for details). Note that only probe sets expressed in a given tissues are considered in this analysis (see Methods). Data from even (Panels A, C, E, G, and I) and odd numbers of remaining probes (Panels B, D, F, H, and J) in probe sets are presented separately due to inherent differences in the way the median polish algorithm employed by RMA processes them. Two-slope red and black lines are provided for the actual and simulated data, respectively (see Methods). The tissue from which the data was collected is indicated in each panel.

Next, we sought to explore broad effects of masking on expression estimates between species. Since we are focusing on 1H_1C probes for expression estimates in both species, a major question concerns the effects of reducing the number of probes in a given probe set on gene expression scores. For this analysis, we applied mask1 to the entire gene expression set (five tissues for all humans and chimpanzees) and calculated the median interquartile range (IQR) of expression scores for all probe sets as a function of the number of remaining probes (Fig. 3, red circles). We considered probe sets comprised of odd and even numbers of remaining probes separately since the RMA median polishing algorithm calculates expression scores from such probe sets slightly differently [35]. This arises from differences in the formulas for determining the median in data sets consisting of odd and even numbers of observations. We propose that the effects of these differences may be enhanced by the small number of probes in each probe set.

As would be expected, the median IQR decreased with an increasing number of probes. However, the relationship was not linear, with a faster decrease occurring when there were five or fewer probes than for probe sets with at least six probes measured. Adjusting for whether the number of probes was odd or even, the change in slope is statistically significant (adjusted *P *< 0.05 for all tissue types). This can be observed from the trend lines (red), generated using a breakpoint at either five probes for the even numbers of probe sets or six for the odd numbers. This reduction in slope supports a requirement of at least six probes in order to have improved stability of the gene expression measure. Similar results were obtained for the corresponding intraspecies IQR comparisons (Additional Files 1 and 2, red circles and lines). The only intraspecies comparison that did not achieve statistical significance at the 0.05 level was for the human testes (adjusted *P *for change in slope = 0.10).

Afterwards, we sought to determine if random probe masking could lead to the observed relationships between median IQR and probes remaining in probe sets. To address this question, we generated a total of nine hundred simulated masked data sets wherein we removed N_1–9 _probes from each of the 4,648 1H_1C_11 probe sets. Overall, this entailed generating one hundred simulated data sets for each N probes removed. We recalculated median interspecies IQRs (Fig. 3, black lines) and median intraspecies IQRs (Additional Files 1 and 2, black lines) for each of these simulated masked data sets. For all tissues, we observed that the median interspecies and intraspecies IQRs derived from these simulated masked data always increase with decreasing numbers of probes within probe sets.

A comparison of lines fit to the simulated data and real masked interspecies data (combining all human and chimpanzee data) separately, found that the estimated slopes vary for three of the five tissues studied (kidney, liver, and testis) (F-test on 2df *P *< 0.05). In contrast, the observed relationships between median IQRs and probe numbers in the actual mask1 brain and heart expression data showed a striking resemblance to the relationships found in the simulated masked brain expression data (Fig. 3A–D).

We sought to address the possibility that factors, such as the rates of evolution, have a substantial influence on the patterns of expression variation observed in different tissues as a function of 1H_1C probe number. For example, it has been demonstrated in the initial analysis of the current data set that patterns of differences in gene expression and gene sequences are similar in humans and chimpanzees [13]. As a first step to approach this issue, we calculated dN/dS ratios for RefSeq transcripts corresponding to approximately 20,000 probe sets in both the human and the chimpanzee lineages (see Additional File 3 and Methods). We chose to analyze dN/dS ratios since they provide a commonly used means of measuring rates of evolution, taking nonsynonymous (dN) and synonymous (dS) substitutions per site into consideration. In bulk, we found that the nucleotide substitution rates of RefSeq transcripts expressed in a given tissue do not significantly vary in relation to the number of 1H_1C probes within the corresponding probe set (Additional File 4). Based on our metrics, the bulk rates of evolution of expressed genes do not explain the discussed relationships between median IQR and probes remaining in probe sets for the five tissues. However, it should be note that these analyses are limited by the quality of current genomic sequence information for mammals such as the common chimpanzee. Thus, this issue could be revisited with improved drafts and annotations of these genomes.

### Effect of probe number on inferences of cross-species differential gene expression

Thereafter, we focused on quantifying the effect(s) that probe number has on inferences of differential expression between humans and chimpanzees. For each tissue, we identified 1H_1C_11 probe sets that showed differential expression across species (see Methods for details). Then, we compared the list of differentially expressed genes in a given simulated data set to the list of differentially expressed genes originally observed in the same tissue. This allowed us to calculate the median and range for each of the following: (i) overlap, (ii) gain, and (iii) loss of inferences of differential expression in the simulated data sets relative to that generated from the original 1H_1C_11 probe sets in each tissue.

**Figure 4:**
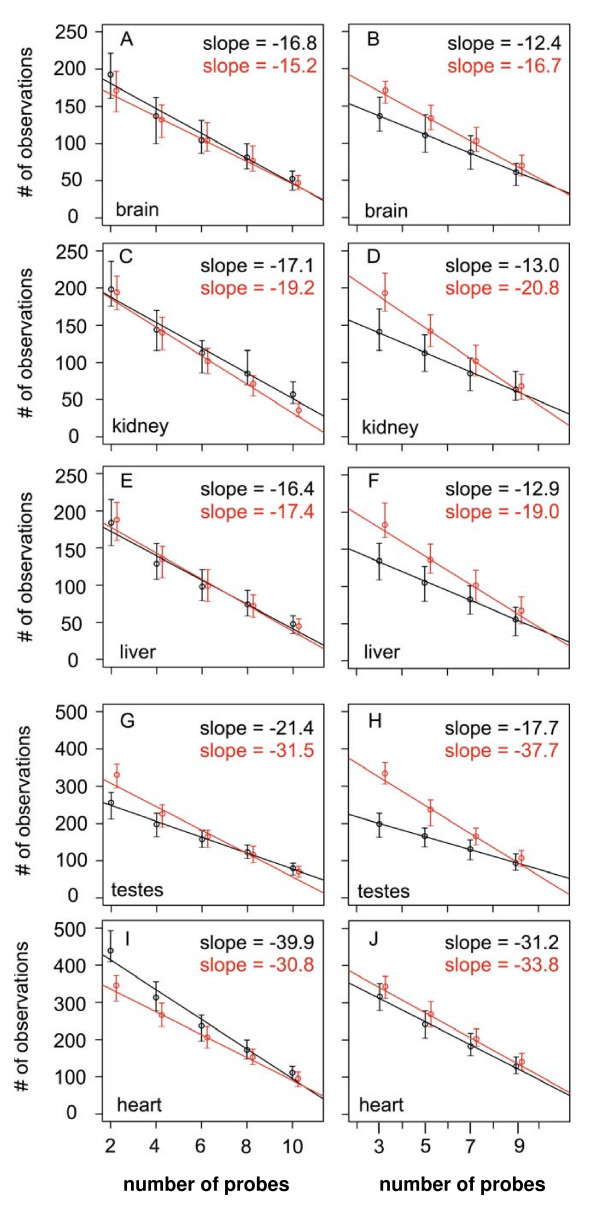
**Effects of probe number on the inferences of differential gene expression**. The median number of gained (black) and lost (red) inferences of differential gene expression (Y-axes) in simulated data sets subjected to random probe masking relative to actual data are plotted against the number of probes in a probe set (X-axes) (see text for details). Error bars represent the observed range of inferred differential gene expression in the simulated data sets. Red and black data points are slightly off-set for visual clarity. Data from probe sets with even (Panels A, C, E, G, and I) and odd numbers of remaining probes (Panels B, D, F, H, and J) are presented separately, as described in Figure 3. The tissue from which the data was collected is indicated in the lower left hand corner of each panel. Note the different scales on the y-axes for heart and testes.

Overall, we observed linear increases in both the gained and lost inferences of differential expression in relationship to decreasing numbers of probes sampled within a probe set (Fig. 4). While this was consistent for all five tissues, probe sets with odd and even numbers of remaining probes behaved slightly differently. Probe sets with even numbers of remaining probes showed more comparable increases in false negative and positive rates with decreasing probe number in all tissues (Fig. 4A, C, E, and 4G), except heart (Fig. 4I). Probe sets with odd numbers of remaining probes showed steeper increases in lost relative to gained inferences with decreasing probe number (Fig. 4B, D, F, H, and 4J).

To illustrate the effects of masking, we compared the inferences of differential gene expression using mask5 (requiring at least 6 1H_1C probes in a probe set) and unmasked data (Additional File 5). For each of the five tissues, the application of the mask drastically reduces inferences of higher expression in humans relative to chimpanzees, as shown by comparing panels A, C, E, G, and I with B, D, F, H, and J. This is consistent with earlier comparative analyses of human and non-human primate transcriptomes which demonstrated that masking was essential to remove false inferences of differential gene expression caused by mismatches between arrayed human probes and non-human primate transcripts (reviewed in ref. [7]).

## Discussion and conclusion

### Strategies for cross-species gene expression analysis

Without appropriate signal processing, the analysis of multi-species gene expression data sets generated using a single oligonucleotide microarray platform can result in heavily biased inferences of differential expression (reviewed in reference [7]). One means to address this problem is to identify individual probes with poor hybridization specificity and sensitivity for any the species compared and remove them from analysis. Ideally, this screening would be performed based on cDNA sequences in all species under consideration. However, for numerous species, such as chimpanzee, publicly available cDNA sequence data is limited. Thus, we conducted our analyses based on the latest releases of the human and chimpanzee genome sequences even though we recognize our inability to query exon-exon junctions or consider polymorphic sequence in either or both species.

However, this computational approach leads to an additional source of error in expression measures due to the variation in the number of probes remaining in a probe set. The interpretation of masked data sets requires an *ad hoc *decision regarding the minimum number of probes required to calculate a gene expression score. Inevitably, some information will be lost in the masking process because some probe sets will need to be excluded when too few probes are remaining to reliably estimate gene expression.

Using simulated data sets comprised of over 4,000 transcripts, we noted that the gained and lost inferences of differential gene expression increased in a linear fashion with decreasing probe number. However, the rates of change were tissue-dependent. The issue of minimal numbers of probes necessary to generate reliable expression scores has been investigated by Lu et al. [28]. Using the related Affymetrix Human Genome U133A platform, they measured differential expression on ten artificial data sets generated from the U133A Latin Square experiment (see ). Based on elegant analyses of IQRs and false discovery rates in control probe sets, interrogating spike-in bacterial and phage transcripts, and human transcript probe sets, the authors concluded that a minimum of four probes is necessary to generate a reliable gene expression score. Although similar in spirit, these analyses cannot be readily compared to the current study due to dramatic differences in experimental design, data processing, and definitions of differential gene expression (2-fold vs. 1.2-fold and a corrected Bayes moderated t-test *P *< 0.05).

Overall, there is strong agreement between the analyses of Lu et al. [28] and the current study that reducing probe number to less than four leads to substantially different results relative to the full complement of 11 probes. This is especially relevant for the analysis of data from oligonucleotide microarrays where only four probes are used to interrogate exon abundance in the human transcriptome, as described in reference [33].

### Conclusions from the empirical data

The data set of Khaitovich and colleagues [13] provides cross-species expression comparisons of the size and complexity necessary to evaluate the effects of probe masking on expression scores in different contexts (i.e. tissues). Based on our comparative analysis of simulated and actual data sets, we found that random probe masking by itself can largely account for the observed relationships between expression variability and the numbers of probes within probe sets in some tissues (i.e. brain), but not others (i.e. testes) (Fig. 3). This implies that in testes, additional factors are strongly influencing the relationships observed in the actual masked data. Given the rapid rates of sequence evolution of fertilization-related genes [36] and relatively slow rates of evolution of brain-expressed genes [37], our observations may reflect relationships between sequence conservation, which can affect the number of 1H_1C probes in a probe set, and expression profiles in human and chimpanzee tissues. However, this is complicated by the fact that a single nucleotide difference between human and chimpanzee transcripts could result in masking all probes in one probe set and no probes in another. Overall, additional investigations into the relationships between the rates of sequence and expression evolution, as first addressed in the initial analysis of the Khaitovich data set [13], are warranted.

In addition, we observed that lost and gained inferences of differential expression across species increased with decreasing numbers of probes in probe sets (Fig. 4). However, it should be noted that these observations are strongly influenced by the criteria used to define differential gene expression. Intriguingly, this relationship was affected by the odd or even nature of the number of remaining probes in a probe sets. Probe sets with even numbers of remaining probes showed comparable increases in the lost and gained inferences of differential expression across species while probe sets with odd numbers of remaining probes showed a skewing towards more lost inference of differential expression than gained. This could be influenced by the way in which RMA uses median polishing methods in data processing [35,38]. Similarly, the choice of criteria and algorithms to identify differentially expressed genes will strongly influence results [39]. As with the normalization RMA platform, we chose the functions available in the limma package for differential gene expression analysis due to widespread use of the package [40,41].

Overall, we conclude that the choice of mask to apply to the human and chimpanzee gene expression data set considered in this study is highly dependent upon the general goals of the meta-analysis. Due to specialized requirements for data quantity and quality, no single mask design is optimal for all applications. Based on our median IQR analyses (Fig. 3, Additional Files 1 and 2), probe sets comprised of 6 or more 1H_1C probes consistently showed similar properties. This metric would provide expression estimates for 89% of the probe sets in the U133Plus2 microarray which have at least 1H_1C probe (i.e. 42,093 of 51,001 probe sets, see Table 2). A slightly less conservative approach would be to apply a mask of requiring 4 or more 1H_1C probes, extrapolating from the analyses described in reference [28]. This metric would provide expression estimates for 93.1% (47,506) of the probe sets with at least 1H_1C probe (Table 2). The relative percentages of probe sets interrogated in these two examples are similar. Thus, we recommend that less stringent masks (mask0, mask1, and mask2) be used for exploratory analyses wherein the number of transcripts with expression scores need to be maximized while more stringent masks (mask3 or higher) be used for more rigorous evaluations of inter- and intraspecies variation.

### Additional applications for masks

In principle, our methods are applicable towards the development of masks for different microarray designs and other cross-species probe masking applications. For example, it is desirable to eliminate probes overlapping common polymorphisms that could affect gene expression data within a given species [42,43]. Likewise, it could be useful to mask probes that overlap splice junctions and thus selectively evaluate the abundance of differentially spliced transcripts. Multiple groups have applied masks to update annotation data and partition out unique sets of probes which each map to a single gene or transcript definition [24-30,44-48]. In the future, mask strategies may be useful for isolating or eliminating the effects of copy number and structural variation on downstream gene expression analyses [49].

## Methods

### Mapping of individual probe sequences to the human and chimpanzee genomes

The Human Genome U133Plus2 microarray (Affymetrix) contains 604,258 perfect match (PM) 25 mer probes organized into 54,675 probe sets which interrogate the relative abundance of ~47,000 human transcripts. For this microarray, 20,112 unique National Center for Biotechnology Information (NCBI) Entrez GeneIDs were mapped to Affymetrix probe designations using the Bioconductor R environment hgu133plus2ENTREZID in the annotation package hgu133plus2.db [50].

Since we used the RMA (robust multi-array average) algorithm [35,38,51], which only considers PM probes in downstream data processing, we discard all information from mismatch (MM) probes, originally designed by Affymetrix to correct for cross-hybridization, from our analysis. For clarity of presentation, we refer to PM probes in the microarray as 'probes'. The probe sequences were obtained from the publicly available NetAffx™ Analysis Center web site [52].

In order to evaluate the probe sequences in the Human Genome U133Plus2 microarray, we developed a new algorithm that quickly and accurately aligns tens of thousands of short sequences to complete genomes or transcriptomes (Renaud and Wolfsberg, manuscript in preparation). This algorithm identifies all sequences with exact matches to a genome (i.e., 100% identity over 100% of the length of the query), and can also be used to identify all sequences that align with a specific number of mismatches. We used this algorithm to align each probe sequence to both strands of the human (NCBI Build 36.1) and chimpanzee genomes (Build 2 Version 1, Oct. 2005 from the Chimpanzee Sequencing and Analysis Consortium). For each probe, it was determined if its sequence matched the human genome zero (0H), one (1H), or multiple times (MH) and whether its sequence matched the chimpanzee genome zero (0C), one (1C), or multiple times (MC) (Table 1, Fig. 1). We define the term 'match' as requiring all 25 bases in a given probe to be identical to a segment of the interrogated genome, that is, we did not allow for any mismatches between the probe and the genome.

Probes were placed into nine distinct categories based on their relationship to both genomes: 0H_0C, 0H_1C, 0H_MC, 1H_0C, 1H_1C, 1H_MC, MH_0C, MH_1C, and MH_MC. Afterwards, each of the 54,675 probe sets was classified based on the abundance of probes within each of these nine categories (Fig. 2).

### Creation of mask files

The cdf environment and the probe environment were downloaded as the R packages hgu133plus2cdf and hgu133plus2probe from the Bioconductor website [50] and installed locally to a defined R libraries directory. Using the C shell (tcsh), the path to the local R libraries directory was specified with the environment variable R_LIBS in the ".cshrc" shell startup script (see Additional File 6). The package CustomCDF was used to remove probes which did not fit the criteria for inclusion in the gene expression summaries from the cdf environment [24,53]. CustomCDF and all R packages used other than those present in the default installation, were installed using biocLite to ensure the installation of all dependencies.

### Application of masks

The Bioconductor package affy [54] was used to import the fifty-five .cel files generated in a previous study of genome-wide expression profiling of human and chimpanzee tissues [13]. The .cel files provide one fluorescent intensity value for each probe in the Human Genome U133Plus2 microarray used in each experiment. These .cel files represent fifty-five gene expression profiling analyses of brain, heart, kidney, liver and testes, each derived from six humans and five chimpanzees (11 total individuals). The function ReadAffy from the R package affy was used resulting in an AffyBatch object. Masking was accomplished using the "removeprobe" function in the R package CustomCDF. The "removeprobe" function alters the cdf environment to create the masked version of the data [24,53].

We generated a series of masks that only retained probes with a single match to both the human and chimpanzee genomes (1H_1C) (see Additional File 6 for detailed instructions and Additional File 7 for all the information necessary to generate these masks). We named these masks based on the number of probe sets that contained more than N probes after initial masking. For example, maskN retains only probe sets with more than N probe(s) remaining after the initial filtering step. Therefore, as mask number (N) increases, the number of probe sets used for interrogating gene expression is reduced (Table 2). Note that the original analyses of the data sets under consideration used an earlier draft of the chimpanzee genome [55] for mask generation [13].

We used the RMA algorithm [35,38] for signal processing and to generate expression values for each set of masked data with the "rma" function in the Bioconductor affy package. Thus, RMA is used to summarize only the probe intensities specified in the modified environment. We report expression measures as log base 2 values. Although we chose RMA as our summarization method, other algorithms could be used. However, caution should be taken when applying algorithms that consider data from mismatch (MM) probes, originally designed by Affymetrix to correct for cross-hybridization in the intended (here, human) transcriptome analyses. In such cases, the mask should also discard data from perfect match (PM)/mismatch (MM) probe pairs where the MM probe now perfectly matches at least sequences in the second species (here, chimpanzee) under consideration, as discussed in reference [32].

### Probe number and variability of expression scores

We used the kOverA function in the Bioconductor genefilter package to select probe sets that interrogated expressed transcripts (herein defined as having a minimum expression criteria of > 100 unit gene expression score in at least four of the five chimpanzees or at least five of the six humans surveyed, unlogged data) in a given tissue of interest. The data were further partitioned so that the expressed transcripts were categorized by the number of probes remaining in their respective probe sets after the initial masking procedure. As a means of surveying variation in gene expression scores, we calculated the median interquartile range (IQR) of the selected probe set as a function of the number of remaining probes for each tissue in (i) humans alone, (ii) chimpanzees alone, and (iii) all humans and all chimpanzees. Mask1 was used for this procedure to prevent the exploration of probe sets with only one probe remaining.

The association between median IQR and number of probes is studied using linear regression. A two-slope line is fit using two predictor variables; the first predictor is the number of probes (2–11) and the second predictor takes on zero when the number of probes is less than the breakpoint and takes on the difference between the number of probes and the breakpoint when the number of probes is larger. A test of the coefficient for the second predictor variable equaling zero is a test of a change in slope for the broken line. In general, one can chose the breakpoint that maximizes the fit of the two lines in terms of variance explained and then test if the slopes for the two lines vary taking into account the multiple testing that occurs in finding the best fit. However, the limited number of probes (five even- or five odd-numbered, excluding a value of one) would not permit us to take advantage of this approach. Instead, we chose five or six as a break point depending on whether we were modeling the even numbered or odd numbered probes. When modeling all probes combined we used a breakpoint of 5.5, and used a covariate to adjust for whether the observation was for a probe set with an even or odd number of probes. The breakpoints used are supported by results from previous papers studying the association between IQR and number of probes [28]. At the same time, if we capture non-linearity through the use of a quadratic term that does not require the specification of a breakpoint, our conclusions remain the same (data not shown). Hypotheses were tested by comparing nested models and computing F-tests. Changes in slopes between simulated and real data are tested by including interaction terms for the two slope predictor variables with a variable denoting data source (simulated or real). Because of the larger number of median IQRs for the simulated data, we chose to model the simulated data using the means of the 100 replicates for each number of probes.

### Probe number and inferences of differential expression

To quantify the effects that the number of probes within a probe set have on inferences of differential expression, we first calculated gene expression scores in all fifty-five .cel files using the mask1 procedure. Afterwards, we identified genes that were differentially expressed across species (herein defined as having a 1.2-fold change, Bayes moderated t-test *P *< 0.05 corrected for multiple comparisons with the Benjamini and Hochberg procedure [56] and a minimum expression criteria of > 100 units in at least four of the five chimpanzees or at least five of the six humans surveyed) using the limma (Linear Models for Microarray Analysis) package [41,57]. We were particularly interested in data obtained from probe sets containing 11 probes (i.e. the number of probes present in a typical U133Plus2 microarray probe set). The designation 1H_1C_11 was used to indicate a perfectly matching set of 11 out of 11 probes. There were 4,648 such 1H_1C_11 probe sets remaining in the masked microarray data.

Next, we generated simulated mask1 files that included all the probes listed in the mask1 file with additional masking of X probes (ranging from one to nine) randomly selected from each 1H_1C_11 probe set. We generated one hundred simulated mask1 files for each value of X. Each of these nine hundred masked data sets was considered in the downstream analysis of gene expression scores for the fifty-five .cel files.

### Probe number and rates of evolution

Rates of nucleotide substitution on the human and chimpanzee lineages were calculated using PAML v. 3.15 [58] and published publicly available multiple sequence alignments of mammalian genes [59], as described in [60]. A total of 19,995 probe sets in the U133Plus2 microarray could be assigned both a single RefSeq ID (based on the publicly available NetAffx™ Analysis Center web site) and a corresponding dN/dS ratio (Additional File 3). This information provided the basis for comparing numbers of 1H_1C probes in probe sets with rates of molecular evolution.

## Authors' contributions

DMT carried out the development and application of the masks, characterized the properties of masked expression data, and helped to draft the manuscript. GR and TGW developed the mapping algorithms, conducted the analyses necessary for assigning the number of times the oligonucleotide probes matched the human and chimpanzee genomes, and helped to draft the manuscript. KDS assisted in characterizing the properties of the masked expression data, provided statistical analyses, and helped to draft the manuscript. MI and DEW conducted the calculations for rates of molecular evolution. JGH conceived of the study, assisted in data analysis, and helped to draft the manuscript. All authors read and approved the final manuscript.

## Supplementary Material

Additional file 1 Effects of probe number on the variation of gene expression scores. The relationships between probe number and the interquartile ranges of human gene expression scores are provided. Click here for file

Additional file 2 Effects of probe number on the variation of gene expression scores. The relationships between probe number and the interquartile ranges of chimpanzee gene expression scores are provided. Click here for file

Additional file 3 Rates of evolution for U133Plus2 microarray probe sets based on their corresponding single RefSeq ID. The rates of evolution for U133Plus2 microarray probe sets based on their corresponding single RefSeq ID are provided. Click here for file

Additional file 4 Relationships between numbers of probes remaining after masking and rates of evolution. The relationships between the number of 1H_1C probes in a probe set and the rates of evolution of corresponding RefSeqs are provided. Click here for file

Additional file 5 Comparative analysis of inferred differential expression based on masked and unmasked data sets. Venn diagrams summarizing the relationships of probe sets showing differential expression based on masked and unmasked data sets. Click here for file

Additional file 6 Instructions for the application of masks for the analysis of human and chimpanzee gene expression data obtained from Affymetrix U133Plus2 microarrays. We provide instructions for the application of masks for the analysis of human and chimpanzee data obtained from Affymetrix U133Plus2 gene expression microarrays. Click here for file

Additional file 7 Master mask file. This is the master mask file that can be applied for cross-species gene expression analyses according to the instructions provided in Additional File 6. Click here for file

## References

[B1] Schena M, Shalon D, Davis RW, Brown PO (1995). Quantitative monitoring of gene expression patterns with a complementary DNA microarray. Science.

[B2] Lockhart DJ, Dong H, Byrne MC, Follettie MT, Gallo MV, Chee MS, Mittmann M, Wang C, Kobayashi M, Horton H (1996). Expression monitoring by hybridization to high-density oligonucleotide arrays. Nat Biotechnol.

[B3] Wodicka L, Dong H, Mittmann M, Ho MH, Lockhart DJ (1997). Genome-wide expression monitoring in Saccharomyces cerevisiae. Nat Biotechnol.

[B4] White KP, Rifkin SA, Hurban P, Hogness DS (1999). Microarray analysis of Drosophila development during metamorphosis. Science.

[B5] Hill AA, Hunter CP, Tsung BT, Tucker-Kellogg G, Brown EL (2000). Genomic analysis of gene expression in C. elegans. Science.

[B6] Schena M, Shalon D, Heller R, Chai A, Brown PO, Davis RW (1996). Parallel human genome analysis: microarray-based expression monitoring of 1000 genes. Proc Natl Acad Sci USA.

[B7] Bar-Or C, Czosnek H, Koltai H (2007). Cross-species microarray hybridizations: a developing tool for studying species diversity. Trends Genet.

[B8] Karaman MW, Houck ML, Chemnick LG, Nagpal S, Chawannakul D, Sudano D, Pike BL, Ho VV, Ryder OA, Hacia JG (2003). Comparative analysis of gene-expression patterns in human and african great ape cultured fibroblasts. Genome Res.

[B9] Enard W, Khaitovich P, Klose J, Zollner S, Heissig F, Giavalisco P, Nieselt-Struwe K, Muchmore E, Varki A, Ravid R (2002). Intra- and interspecific variation in primate gene expression patterns. Science.

[B10] Caceres M, Lachuer J, Zapala MA, Redmond JC, Kudo L, Geschwind DH, Lockhart DJ, Preuss TM, Barlow C (2003). Elevated gene expression levels distinguish human from non-human primate brains. Proc Natl Acad Sci USA.

[B11] Uddin M, Wildman DE, Liu G, Xu W, Johnson RM, Hof PR, Kapatos G, Grossman LI, Goodman M (2004). Sister grouping of chimpanzees and humans as revealed by genome-wide phylogenetic analysis of brain gene expression profiles. Proc Natl Acad Sci USA.

[B12] Khaitovich P, Muetzel B, She X, Lachmann M, Hellmann I, Dietzsch J, Steigele S, Do HH, Weiss G, Enard W (2004). Regional patterns of gene expression in human and chimpanzee brains. Genome Res.

[B13] Khaitovich P, Hellmann I, Enard W, Nowick K, Leinweber M, Franz H, Weiss G, Lachmann M, Paabo S (2005). Parallel patterns of evolution in the genomes and transcriptomes of humans and chimpanzees. Science.

[B14] Calarco JA, Xing Y, Caceres M, Calarco JP, Xiao X, Pan Q, Lee C, Preuss TM, Blencowe BJ (2007). Global analysis of alternative splicing differences between humans and chimpanzees. Genes Dev.

[B15] Hacia JG, Fan JB, Ryder O, Jin L, Edgemon K, Ghandour G, Mayer RA, Sun B, Hsie L, Robbins CM (1999). Determination of ancestral alleles for human single-nucleotide polymorphisms using high-density oligonucleotide arrays. Nat Genet.

[B16] Hacia JG, Makalowski W, Edgemon K, Erdos MR, Robbins CM, Fodor SP, Brody LC, Collins FS (1998). Evolutionary sequence comparisons using high-density oligonucleotide arrays. Nat Genet.

[B17] Blekhman R, Oshlack A, Chabot AE, Smyth GK, Gilad Y (2008). Gene regulation in primates evolves under tissue-specific selection pressures. PLoS Genet.

[B18] Gilad Y, Rifkin SA, Bertone P, Gerstein M, White KP (2005). Multi-species microarrays reveal the effect of sequence divergence on gene expression profiles. Genome Res.

[B19] Oshlack A, Chabot AE, Smyth GK, Gilad Y (2007). Using DNA microarrays to study gene expression in closely related species. Bioinformatics.

[B20] Kurtz S, Phillippy A, Delcher AL, Smoot M, Shumway M, Antonescu C, Salzberg SL (2004). Versatile and open software for comparing large genomes. Genome Biol.

[B21] Doring A, Weese D, Rausch T, Reinert K (2008). SeqAn an efficient, generic C++ library for sequence analysis. BMC Bioinformatics.

[B22] Liu H, Zeeberg BR, Qu G, Koru AG, Ferrucci A, Kahn A, Ryan MC, Nuhanovic A, Munson PJ, Reinhold WC (2007). AffyProbeMiner: a web resource for computing or retrieving accurately redefined Affymetrix probe sets. Bioinformatics.

[B23] Carter SL, Eklund AC, Mecham BH, Kohane IS, Szallasi Z (2005). Redefinition of affymetrix probe sets by sequence overlap with cDNA microarray probes reduces cross-platform inconsistencies in cancer-associated gene expression measurements. Bmc Bioinformatics.

[B24] Dai M, Wang P, Boyd AD, Kostov G, Athey B, Jones EG, Bunney WE, Myers RM, Speed TP, Akil H (2005). Evolving gene/transcript definitions significantly alter the interpretation of GeneChip data. Nucleic Acids Res.

[B25] Gautier L, Moller M, Friis-Hansen L, Knudsen S (2004). Alternative mapping of probes to genes for Affymetrix chips. BMC Bioinformatics.

[B26] Kong SW, Hwang KB, Kim RD, Zhang BT, Greenberg SA, Kohane IS, Park PJ (2005). CrossChip: a system supporting comparative analysis of different generations of Affymetrix arrays. Bioinformatics.

[B27] Sandberg R, Larsson O (2007). Improved precision and accuracy for microarrays using updated probe set definitions. BMC Bioinformatics.

[B28] Lu J, Lee JC, Salit ML, Cam MC (2007). Transcript-based redefinition of grouped oligonucleotide probe sets using AceView: high-resolution annotation for microarrays. BMC Bioinformatics.

[B29] Elo LL, Lahti L, Skottman H, Kylaniemi M, Lahesmaa R, Aittokallio T (2005). Integrating probe-level expression changes across generations of Affymetrix arrays. Nucleic Acids Res.

[B30] Mecham BH, Wetmore DZ, Szallasi Z, Sadovsky Y, Kohane I, Mariani TJ (2004). Increased measurement accuracy for sequence-verified microarray probes. Physiol Genomics.

[B31] Oldham MC, Horvath S, Geschwind DH (2006). Conservation and evolution of gene coexpression networks in human and chimpanzee brains. Proc Natl Acad Sci USA.

[B32] Nagpal S, Karaman MW, Timmerman MM, Ho VV, Pike BL, Hacia JG (2004). Improving the sensitivity and specificity of gene expression analysis in highly related organisms through the use of electronic masks. Nucleic Acids Res.

[B33] Robinson MD, Speed TP (2007). A comparison of Affymetrix gene expression arrays. BMC Bioinformatics.

[B34] Okoniewski MJ, Hey Y, Pepper SD, Miller CJ (2007). High correspondence between Affymetrix exon and standard expression arrays. Biotechniques.

[B35] Irizarry RA, Bolstad BM, Collin F, Cope LM, Hobbs B, Speed TP (2003). Summaries of Affymetrix GeneChip probe level data. Nucleic Acids Res.

[B36] Gibbs RA, Rogers J, Katze MG, Bumgarner R, Weinstock GM, Mardis ER, Remington KA, Strausberg RL, Venter JC, Wilson RK (2007). Evolutionary and biomedical insights from the rhesus macaque genome. Science.

[B37] Wang HY, Chien HC, Osada N, Hashimoto K, Sugano S, Gojobori T, Chou CK, Tsai SF, Wu CI, Shen CK (2007). Rate of evolution in brain-expressed genes in humans and other primates. PLoS Biol.

[B38] Irizarry RA, Hobbs B, Collin F, Beazer-Barclay YD, Antonellis KJ, Scherf U, Speed TP (2003). Exploration, normalization, and summaries of high density oligonucleotide array probe level data. Biostatistics.

[B39] Choe SE, Boutros M, Michelson AM, Church GM, Halfon MS (2005). Preferred analysis methods for Affymetrix GeneChips revealed by a wholly defined control dataset. Genome Biol.

[B40] Xia X, McClelland M, Wang Y (2005). WebArray: an online platform for microarray data analysis. BMC Bioinformatics.

[B41] Smyth G, Gentleman RVC, Dudoit S, Irizarry R, Huber W (2005). Limma: linear models for microarray data. Bioinformatics and Computational Biology Solutions using R and Bioconductor.

[B42] Benovoy D, Kwan T, Majewski J (2008). Effect of polymorphisms within probe-target sequences on olignonucleotide microarray experiments. Nucleic Acids Res.

[B43] Kumari S, Verma LK, Weller JW (2007). AffyMAPSDetector: a software tool to characterize Affymetrix GeneChip expression arrays with respect to SNPs. BMC Bioinformatics.

[B44] Ferrari F, Bortoluzzi S, Coppe A, Sirota A, Safran M, Shmoish M, Ferrari S, Lancet D, Danieli GA, Bicciato S (2007). Novel definition files for human GeneChips based on GeneAnnot. BMC Bioinformatics.

[B45] Lee JC, Stiles D, Lu J, Cam MC (2007). A detailed transcript-level probe annotation reveals alternative splicing based microarray platform differences. BMC Genomics.

[B46] Chalifa-Caspi V, Yanai I, Ophir R, Rosen N, Shmoish M, Benjamin-Rodrig H, Shklar M, Stein TI, Shmueli O, Safran M (2004). GeneAnnot: comprehensive two-way linking between oligonucleotide array probesets and GeneCards genes. Bioinformatics.

[B47] Hwang KB, Kong SW, Greenberg SA, Park PJ (2004). Combining gene expression data from different generations of oligonucleotide arrays. BMC Bioinformatics.

[B48] Alberts R, Terpstra P, Hardonk M, Bystrykh LV, de Haan G, Breitling R, Nap JP, Jansen RC (2007). A verification protocol for the probe sequences of Affymetrix genome arrays reveals high probe accuracy for studies in mouse, human and rat. BMC Bioinformatics.

[B49] Cooper GM, Nickerson DA, Eichler EE (2007). Mutational and selective effects on copy-number variants in the human genome. Nat Genet.

[B50] Gentleman RC, Carey VJ, Bates DM, Bolstad B, Dettling M, Dudoit S, Ellis B, Gautier L, Ge Y, Gentry J (2004). Bioconductor: open software development for computational biology and bioinformatics. Genome Biol.

[B51] Bolstad BM, Irizarry RA, Astrand M, Speed TP (2003). A comparison of normalization methods for high density oligonucleotide array data based on variance and bias. Bioinformatics.

[B52] Liu G, Loraine AE, Shigeta R, Cline M, Cheng J, Valmeekam V, Sun S, Kulp D, Siani-Rose MA (2003). NetAffx: Affymetrix probesets and annotations. Nucleic Acids Res.

[B53] Dai M, Wang P, Jakupovic E, Watson SJ, Meng F (2007). Web-based GeneChip analysis system for large-scale collaborative projects. Bioinformatics.

[B54] Gautier L, Cope L, Bolstad BM, Irizarry RA (2004). affy – analysis of Affymetrix GeneChip data at the probe level. Bioinformatics.

[B55] Consortium TCSaA (2005). Initial sequence of the chimpanzee genome and comparison with the human genome. Nature.

[B56] Benjamini Y, Hochberg Y (1995). Controlling the False Discovery Rate – a Practical and Powerful Approach to Multiple Testing. Journal of the Royal Statistical Society Series B-Methodological.

[B57] Smyth GK (2004). Linear models and empirical bayes methods for assessing differential expression in microarray experiments. Stat Appl Genet Mol Biol.

[B58] Yang Z (1997). PAML: a program package for phylogenetic analysis by maximum likelihood. Comput Appl Biosci.

[B59] Liu G, Uddin M, Islam M, Goodman M, Grossman LI, Romero R, Wildman DE (2007). OCPAT: an online codon-preserved alignment tool for evolutionary genomic analysis of protein coding sequences. Source Code Biol Med.

[B60] Uddin M, Goodman M, Erez O, Romero R, Liu G, Islam M, Opazo JC, Sherwood CC, Grossman LI, Wildman DE (2008). Distinct genomic signatures of adaptation in pre- and postnatal environments during human evolution. Proc Natl Acad Sci USA.

