# Supplementary material for: Development and evaluation of new mask protocols for gene expression profiling in humans and chimpanzees
Source: BMC Bioinformatics. 2009 Mar 5;10:77. doi: 10.1186/1471-2105-10-77 (PMC2660304; doi:10.1186/1471-2105-10-77)
Supplement: Additional file 1 — Effects of probe number on the variation of gene expression scores. The relationships between probe number and the interquartile ranges of human gene expression scores are provided. [file 1471-2105-10-77-S1.ppt]

## Slide 1
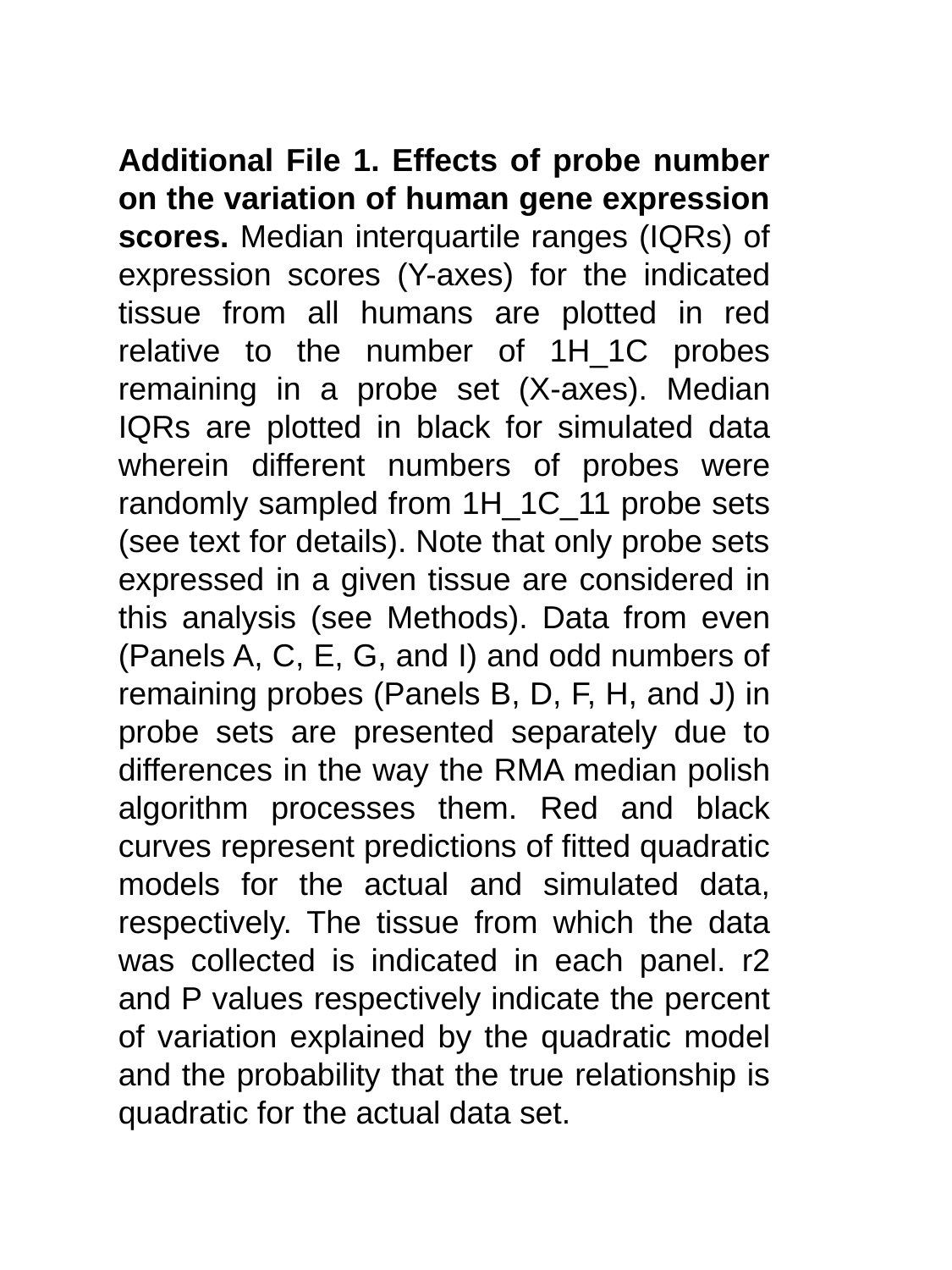

Additional File 1. Effects of probe number on the variation of human gene expression scores. Median interquartile ranges (IQRs) of expression scores (Y-axes) for the indicated tissue from all humans are plotted in red relative to the number of 1H_1C probes remaining in a probe set (X-axes). Median IQRs are plotted in black for simulated data wherein different numbers of probes were randomly sampled from 1H_1C_11 probe sets (see text for details). Note that only probe sets expressed in a given tissue are considered in this analysis (see Methods). Data from even (Panels A, C, E, G, and I) and odd numbers of remaining probes (Panels B, D, F, H, and J) in probe sets are presented separately due to differences in the way the RMA median polish algorithm processes them. Red and black curves represent predictions of fitted quadratic models for the actual and simulated data, respectively. The tissue from which the data was collected is indicated in each panel. r2 and P values respectively indicate the percent of variation explained by the quadratic model and the probability that the true relationship is quadratic for the actual data set.

## Slide 2
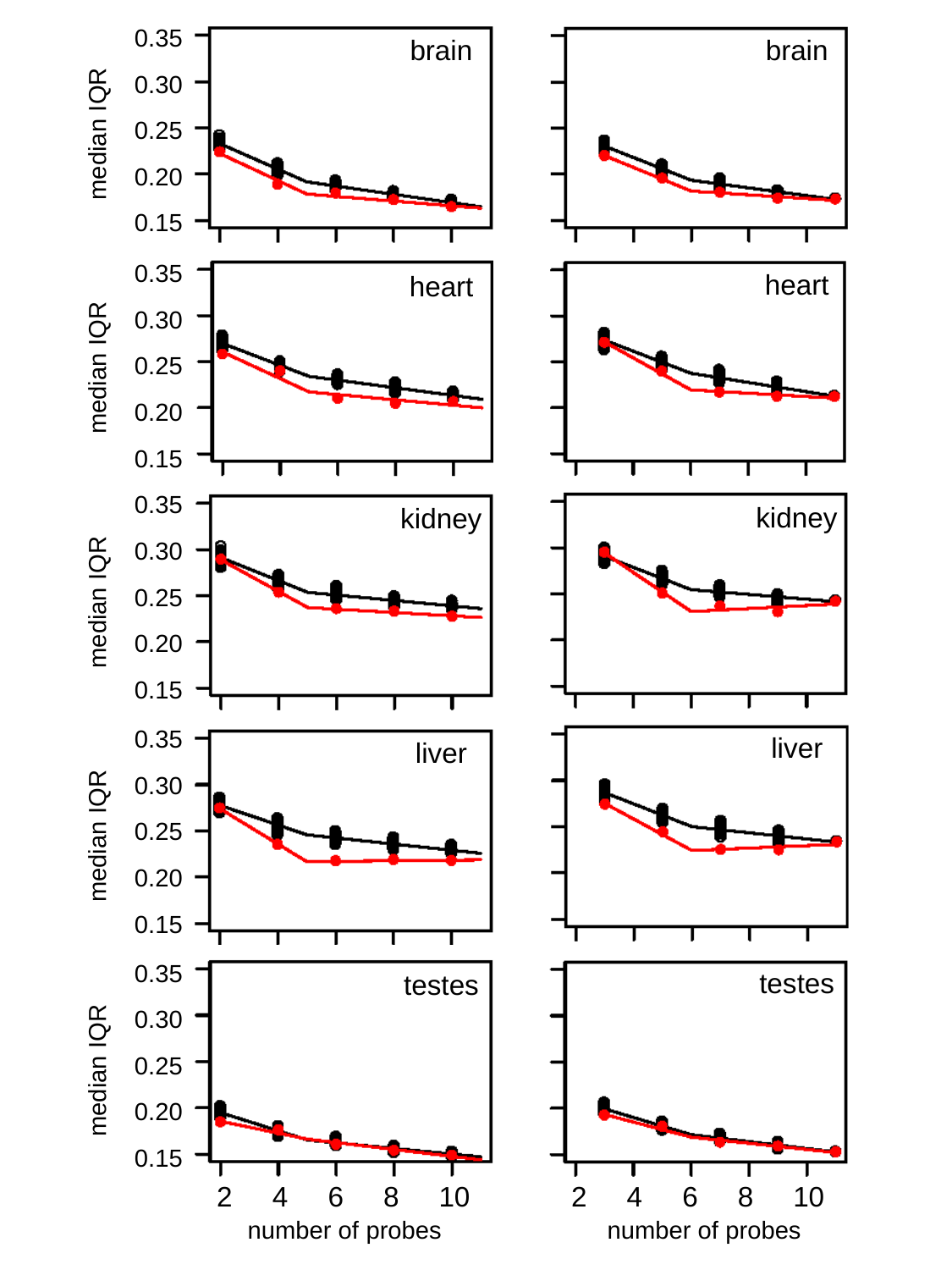

0.35
0.30
0.25
0.20
0.15
brain
brain
median IQR
0.35
0.30
0.25
0.20
0.15
heart
heart
median IQR
0.35
0.30
0.25
0.20
0.15
kidney
kidney
median IQR
0.35
0.30
0.25
0.20
0.15
liver
liver
median IQR
0.35
0.30
0.25
0.20
0.15
testes
testes
median IQR
2
4
6
8
10
2
4
6
8
10
number of probes
number of probes
