# Supplementary material for: Development and evaluation of new mask protocols for gene expression profiling in humans and chimpanzees
Source: BMC Bioinformatics. 2009 Mar 5;10:77. doi: 10.1186/1471-2105-10-77 (PMC2660304; doi:10.1186/1471-2105-10-77)
Supplement: Additional file 4 — Relationships between numbers of probes remaining after masking and rates of evolution. The relationships between the number of 1H_1C probes in a probe set and the rates of evolution of corresponding RefSeqs are provided. [file 1471-2105-10-77-S4.ppt]

## Slide 1
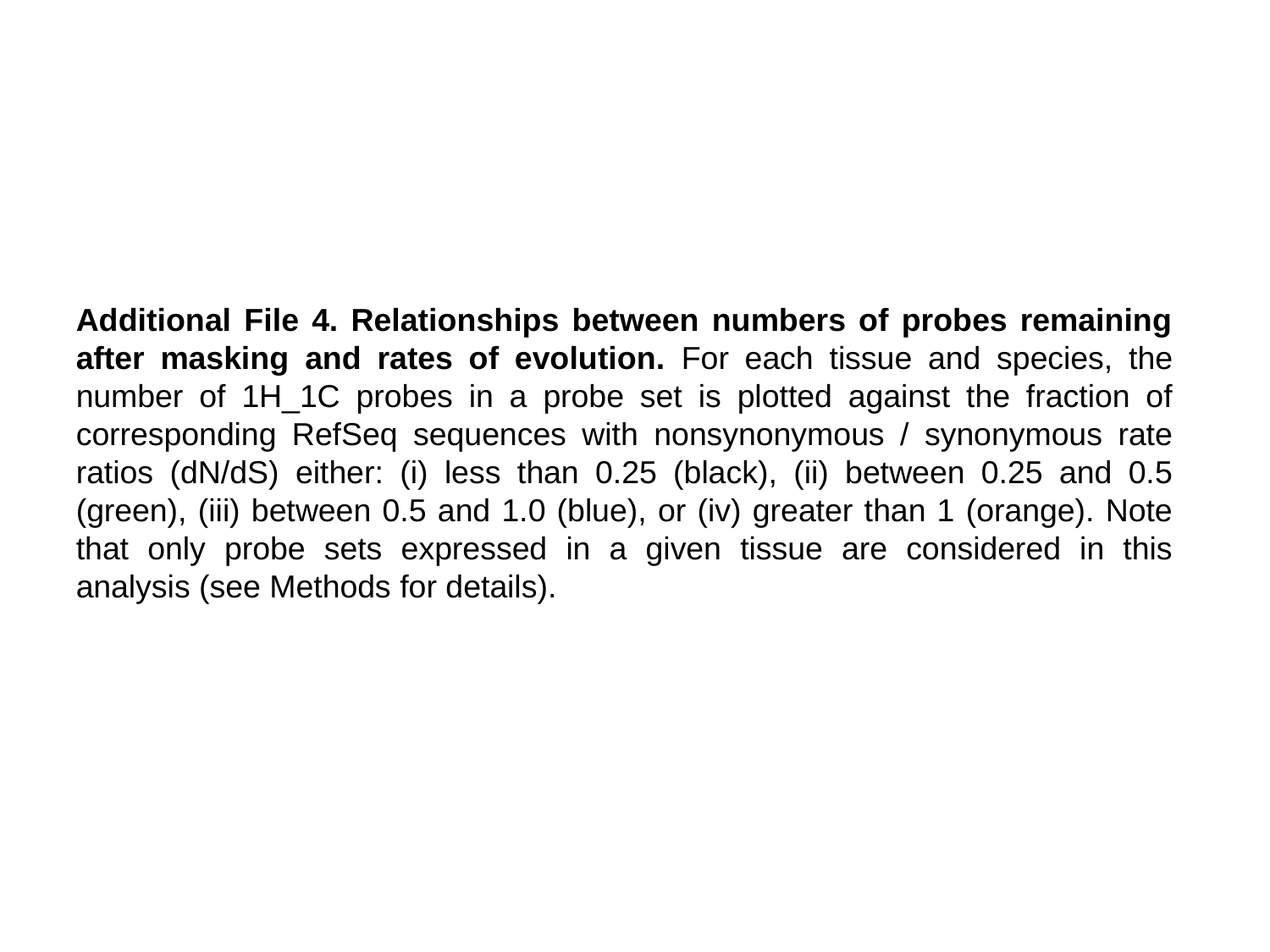

Additional File 4. Relationships between numbers of probes remaining after masking and rates of evolution. For each tissue and species, the number of 1H_1C probes in a probe set is plotted against the fraction of corresponding RefSeq sequences with nonsynonymous / synonymous rate ratios (dN/dS) either: (i) less than 0.25 (black), (ii) between 0.25 and 0.5 (green), (iii) between 0.5 and 1.0 (blue), or (iv) greater than 1 (orange). Note that only probe sets expressed in a given tissue are considered in this analysis (see Methods for details).

## Slide 2
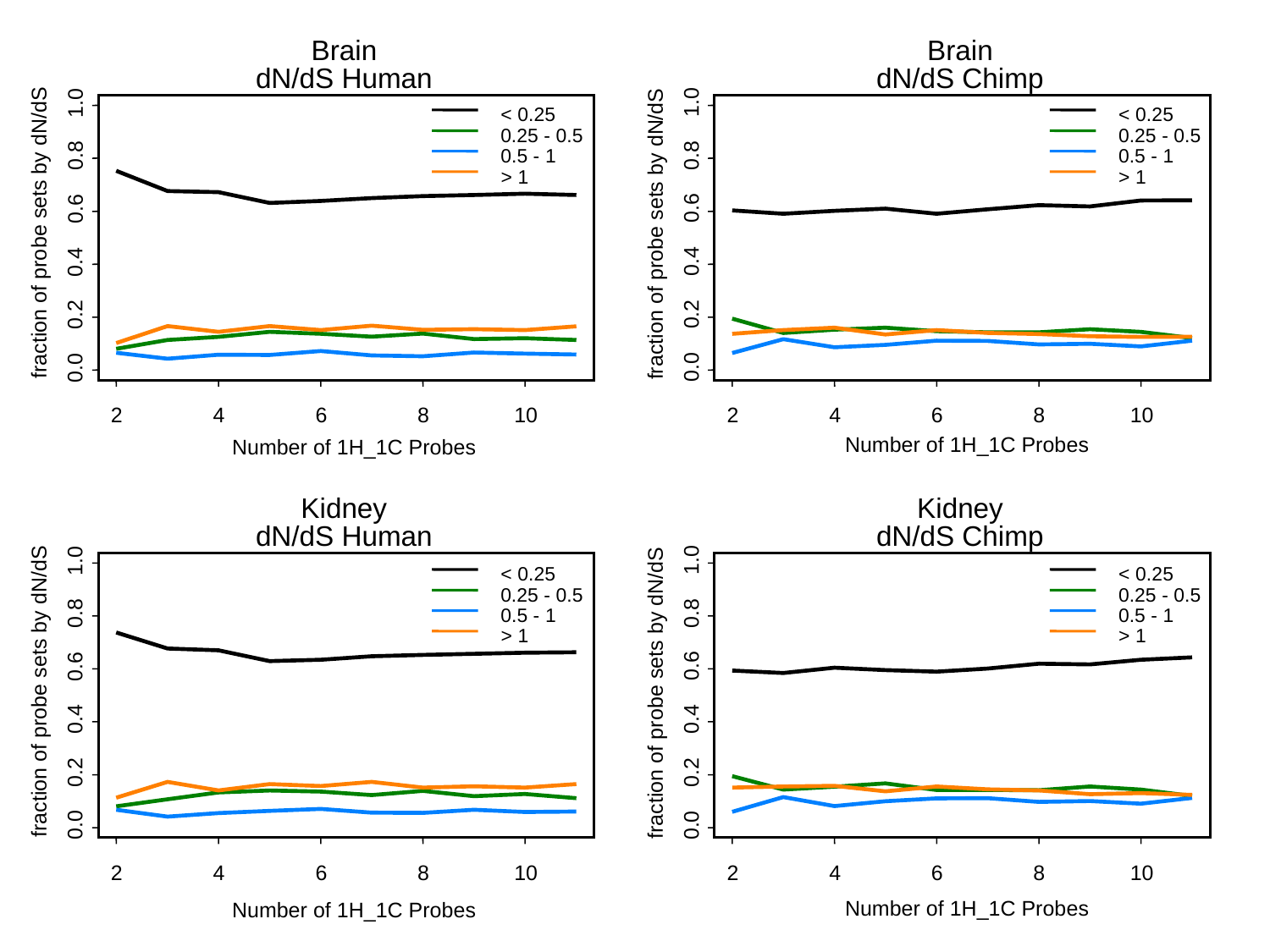

Brain
Brain
dN/dS Human
dN/dS Chimp
1.0
1.0
< 0.25
< 0.25
0.25 - 0.5
0.25 - 0.5
0.8
0.8
0.5 - 1
0.5 - 1
> 1
> 1
0.6
0.6
fraction of probe sets by dN/dS
fraction of probe sets by dN/dS
0.4
0.4
0.2
0.2
0.0
0.0
2
4
6
8
10
2
4
6
8
10
Number of 1H_1C Probes
Number of 1H_1C Probes
Kidney
Kidney
dN/dS Human
dN/dS Chimp
1.0
1.0
< 0.25
< 0.25
0.25 - 0.5
0.25 - 0.5
0.8
0.8
0.5 - 1
0.5 - 1
> 1
> 1
0.6
0.6
fraction of probe sets by dN/dS
fraction of probe sets by dN/dS
0.4
0.4
0.2
0.2
0.0
0.0
2
4
6
8
10
2
4
6
8
10
Number of 1H_1C Probes
Number of 1H_1C Probes

## Slide 3
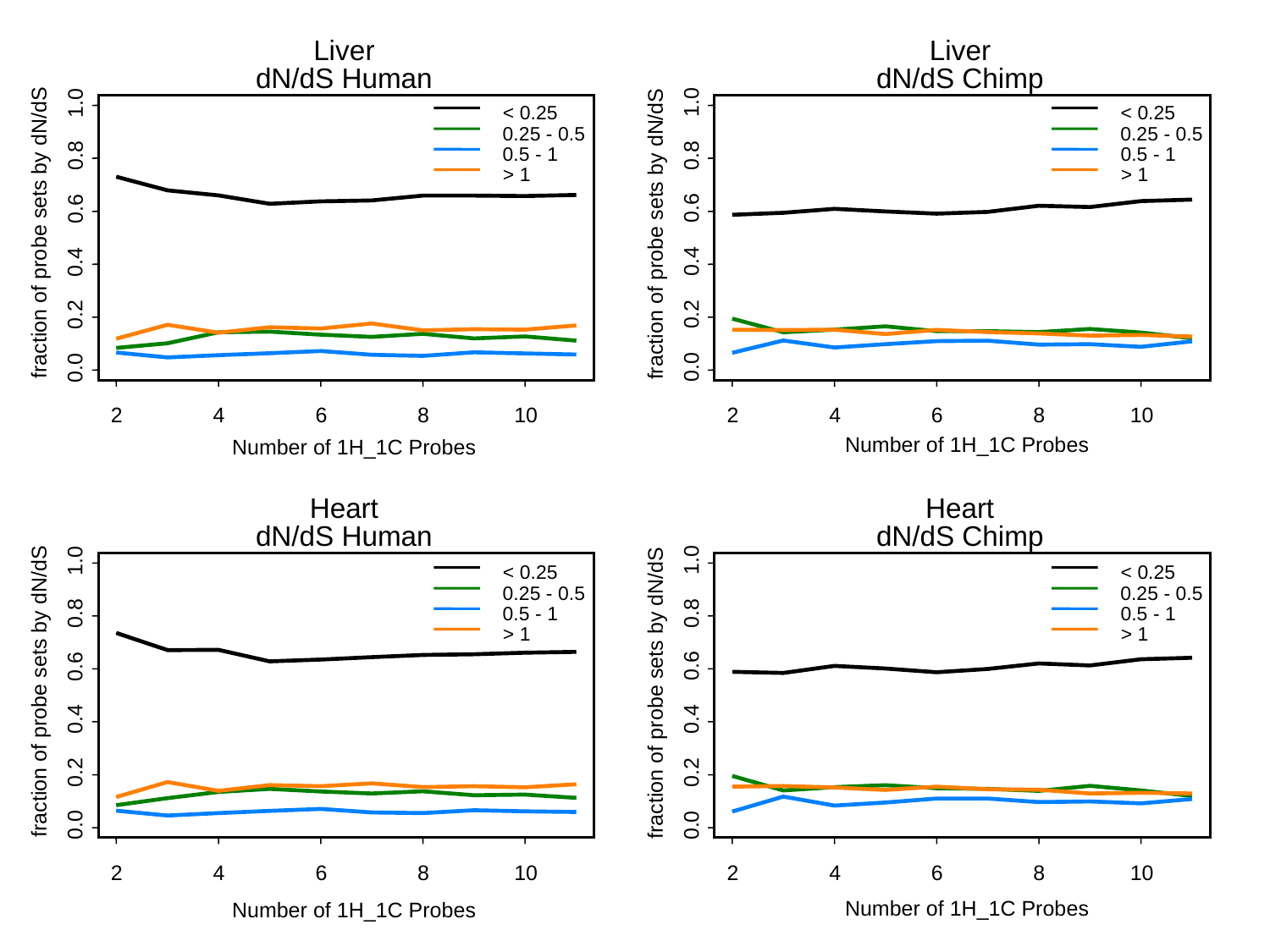

Liver
Liver
dN/dS Human
dN/dS Chimp
1.0
1.0
< 0.25
< 0.25
0.25 - 0.5
0.25 - 0.5
0.5 - 1
0.5 - 1
0.8
0.8
> 1
> 1
0.6
0.6
fraction of probe sets by dN/dS
fraction of probe sets by dN/dS
0.4
0.4
0.2
0.2
0.0
0.0
2
4
6
8
10
2
4
6
8
10
Number of 1H_1C Probes
Number of 1H_1C Probes
Heart
Heart
dN/dS Human
dN/dS Chimp
1.0
1.0
< 0.25
< 0.25
0.25 - 0.5
0.25 - 0.5
0.8
0.8
0.5 - 1
0.5 - 1
> 1
> 1
0.6
0.6
fraction of probe sets by dN/dS
fraction of probe sets by dN/dS
0.4
0.4
0.2
0.2
0.0
0.0
2
4
6
8
10
2
4
6
8
10
Number of 1H_1C Probes
Number of 1H_1C Probes

## Slide 4
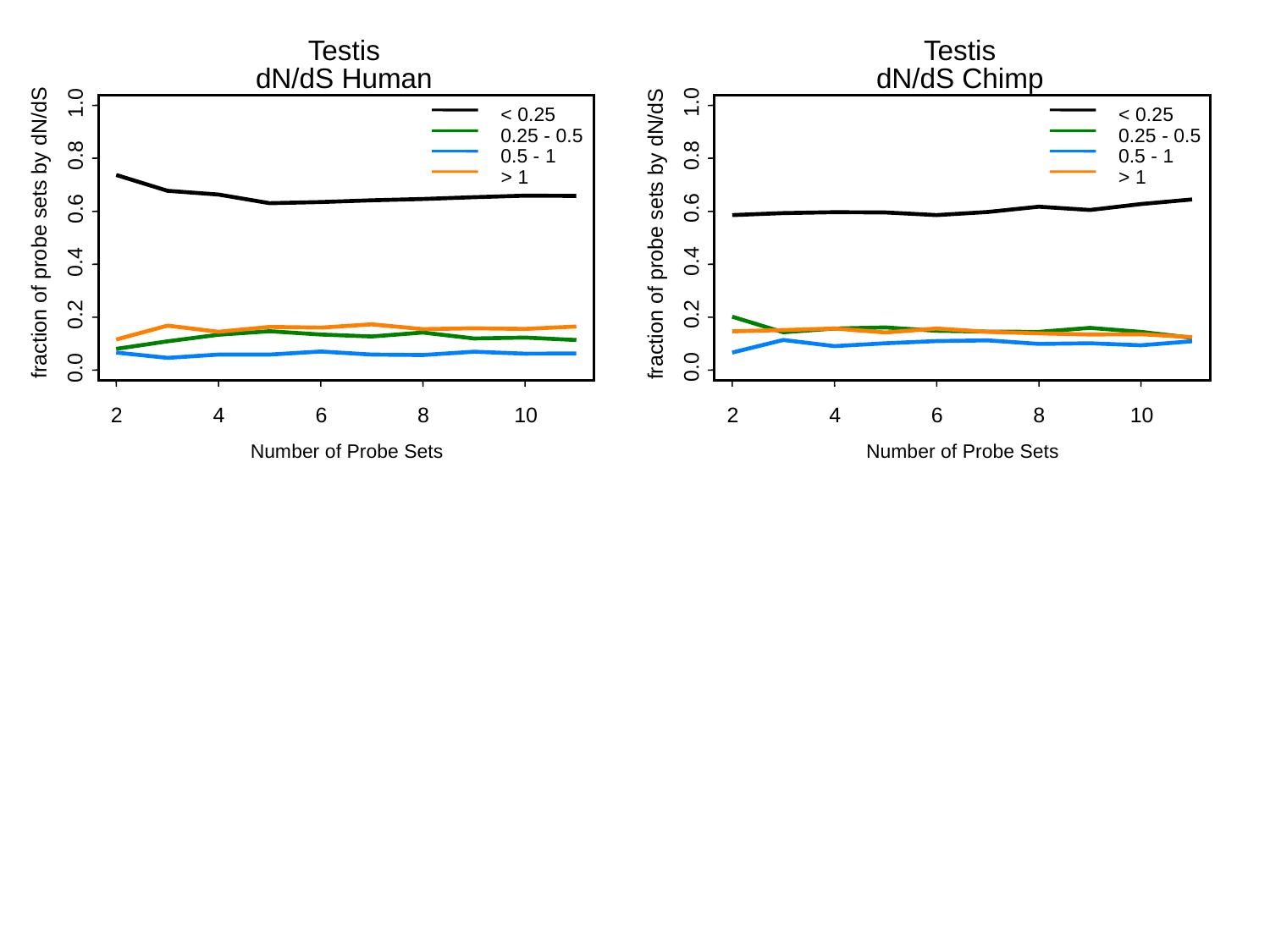

Testis
Testis
dN/dS Human
dN/dS Chimp
1.0
1.0
< 0.25
< 0.25
0.25 - 0.5
0.25 - 0.5
0.8
0.8
0.5 - 1
0.5 - 1
> 1
> 1
0.6
0.6
fraction of probe sets by dN/dS
fraction of probe sets by dN/dS
0.4
0.4
0.2
0.2
0.0
0.0
2
4
6
8
10
2
4
6
8
10
Number of Probe Sets
Number of Probe Sets
