# Supplementary material for: Development and evaluation of new mask protocols for gene expression profiling in humans and chimpanzees
Source: BMC Bioinformatics. 2009 Mar 5;10:77. doi: 10.1186/1471-2105-10-77 (PMC2660304; doi:10.1186/1471-2105-10-77)
Supplement: Additional file 5 — Comparative analysis of inferred differential expression based on masked and unmasked data sets. Venn diagrams summarizing the relationships of probe sets showing differential expression based on masked and unmasked data sets. [file 1471-2105-10-77-S5.ppt]

## Slide 1
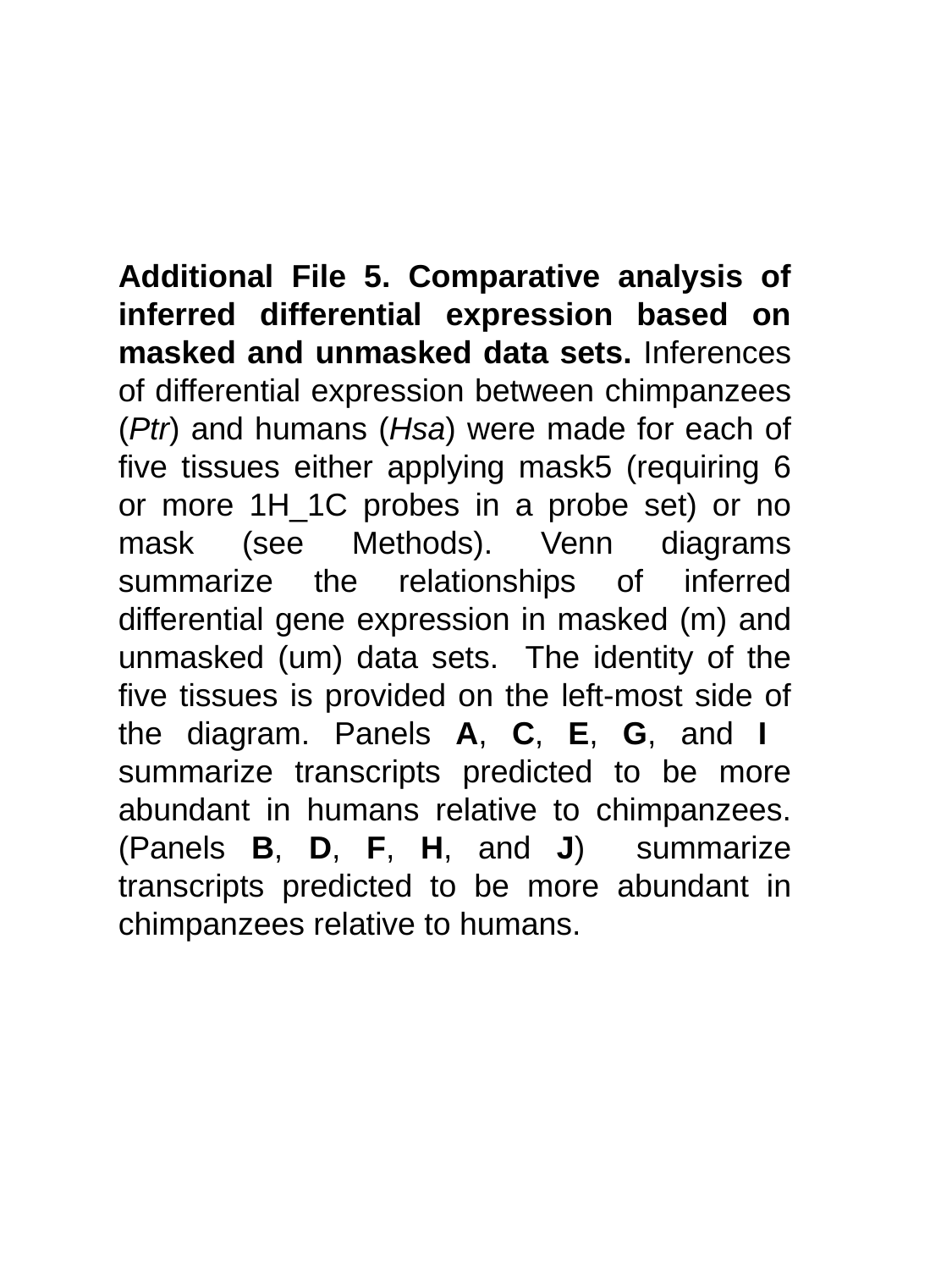

Additional File 5. Comparative analysis of inferred differential expression based on masked and unmasked data sets. Inferences of differential expression between chimpanzees (Ptr) and humans (Hsa) were made for each of five tissues either applying mask5 (requiring 6 or more 1H_1C probes in a probe set) or no mask (see Methods). Venn diagrams summarize the relationships of inferred differential gene expression in masked (m) and unmasked (um) data sets. The identity of the five tissues is provided on the left-most side of the diagram. Panels A, C, E, G, and I summarize transcripts predicted to be more abundant in humans relative to chimpanzees. (Panels B, D, F, H, and J) summarize transcripts predicted to be more abundant in chimpanzees relative to humans.

## Slide 2
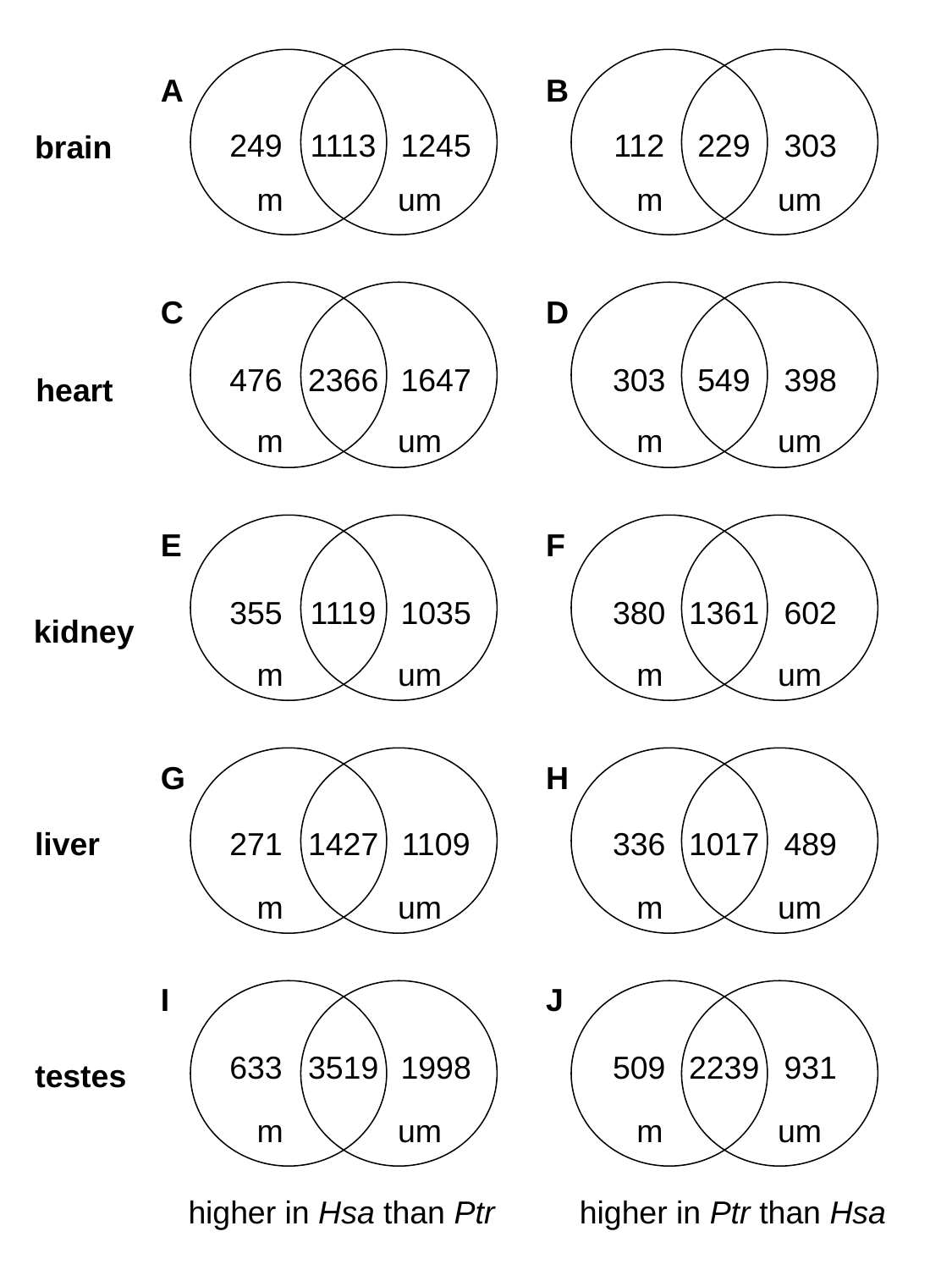

A
B
249
1113
1245
112
229
303
brain
m
um
m
um
C
D
476
2366
1647
303
549
398
heart
m
um
m
um
E
F
355
1119
1035
380
1361
602
kidney
m
um
m
um
G
H
liver
271
1427
1109
336
1017
489
m
um
m
um
I
J
633
3519
1998
509
2239
931
testes
m
um
m
um
higher in Hsa than Ptr
higher in Ptr than Hsa
