# Supplementary material for: Development and evaluation of new mask protocols for gene expression profiling in humans and chimpanzees
Source: BMC Bioinformatics. 2009 Mar 5;10:77. doi: 10.1186/1471-2105-10-77 (PMC2660304; doi:10.1186/1471-2105-10-77)
Supplement: Additional file 6 — Instructions for the application of masks for the analysis of human and chimpanzee gene expression data obtained from Affymetrix U133Plus2 microarrays. We provide instructions for the application of masks for the analysis of human and chimpanzee data obtained from Affymetrix U133Plus2 gene expression microarrays. [file 1471-2105-10-77-S6.doc]

**Additional File 6. Instructions for the application of masks for the analysis of human and chimpanzee gene expression data obtained from Affymetrix U133Plus2 microarrays.**

First, we provide a simple tutorial to guide the user through the process of install and setting up the R packages necessary for mask analysis. This involves steps 1-6 which will only need to be done once.

Second, we detail the steps necessary to create masked U133Plus2 gene expression data. We provide an example of applying these masks to 55 .cel files, which would correspond to the analyses described in the main text. This involves steps 7-11.

**Simple tutorial for the installation of R packages (Steps 1-6)**

**1. Find your local Linux server and obtain an account.**

Using a tool such as Terminal for a Macintosh computer or a terminal emulator in Windows such as PuTTY

(or any X-windows tunnel that supports ssh)

Connect to your Linux or Unix server with ssh.

These directions assume that R is installed on the server.

In these instructions the $ is the Unix prompt and should not be typed.

The > is the R prompt you will see when you use R. It also should not be typed.

**2. Make a directory for all of your R libraries.**

You may not have permissions to install special R libraries at a system-wide level, so you will need a place to install locally.

Go to the appropriate directory such as your home directory and type:

$ mkdir R_libraries

For making this directory in the home directory:

$ mkdir ~/R_libraries

To double check where you just put this directory, type the following:

$ cd R_libraries

or if you made the directory by the second method use:

$ cd ~/R_libraries

The above command is to “change directory” it will change into the directory you just made.

Use the following command:

$ ls

This will list the files in the directory. If you just created the directory it will be empty.

$ pwd

This will display the full path of the directory you created.

Make note of the path to the directory. You will need to know this path for subsequent steps.

**3. Give instructions for R to always find your specific R libraries.**

Your Linux account usually makes use of either tcsh shell or bash shell.

To learn which Unix shell you are using type echo $SHELL at the $ prompt.

a. for tcsh shell:

Type the following in your home directory:

$ emacs .cshrc

The program emacs will allow you to edit the .cshrc file.

(Use any text editor you want but BE CAREFUL with the file!)

create or modify the .cshrc file with the following line:

setenv R_LIBS /home/full_path/to_your_local/R_library_directory_name

Ctrl X S keys all at the same time will save the changes.

Ctrl X C keys all at the same time will exit emacs.

Then add this line without erasing anything else you may find in the file:

setenv R_LIBS /home/full_path/to_your_local/R_library_directory_name

for bash shell:

Create or modify the .bashrc file starting with the following:

$ emacs .bashrc

An editor allows you to edit the .bashrc file. (BE CAREFUL with the file!)

Then add this line without erasing anything else you may find in the file:

export R_LIBS=/home/full_path/to_your_local/R_library_directory_name

Ctrl X S keys all at the same time will save the changes.

Ctrl X C keys all at the same time will exit emacs.

You may have to log out and back into your account for the .bashrc file to be recognized.

If you do not want to risk changing the .bashrc or the .cshrc files

type the appropriate command,

either

$ setenv R_LIBS full/path

or

$ export R_LIBS=full/path

each time you log in to your system.

If these instructions do not prove useful, create a .Renviron file instead.

Create a file in your home directory called .Renviron with the following line in the file

R_LIBS= that/same_path/to/the_library

**4. Install Bioconductor if necessary.**

Start an R session:

$ R

direct R to the updated Bioconductor page (you will need an internet connection):

> source("http://bioconductor.org/biocLite.R")

Run biocLite. This is a mechanism for installing R packages properly.

> biocLite()

**5. Load the specific cdf package that you need.**

biocLite("hgu133plus2cdf") or the appropriate package for your microarray platform.

A cdf environment can be made from Affymetrix cdf files.

See package makecdfenv for further information about making cdf environments.

The cdf environment is a data structure. It can be used by the Bioconductor affy package.

For the hgu133plus2 microarray the package including the cdf environment is publicly available.

Many pre-assembled packages include cdf environments.

Please visit http://www.bioconductor.org/ to check for package availability for your specific Affymetrix microarray

**6. Download and install the CustomCDF package. This is what you will use to apply the mask.**

The package called CustomCDF by Manhong Dai can be found at:

http://arrayanalysis.mbni.med.umich.edu/MBNIUM.html#CustomCDF

1. Download the .tar.gz version
2. Place the file in your home directory where you have your Linux account.

One easy tool for moving files is winscp.

c. While in the home directory, type:

$ R CMD INSTALL -l where_to_install_path packagename.tar.gz

For example:

$ R CMD INSTALL -l /home/folder_for_my_R_packages CustomCDF.tar.gz

If you did everything else in the previous steps you should be able to give the INSTALL command without the path. For example:

$ R CMD INSTALL CustomCDF.tar.gz

Notice you do this at the Unix prompt and not in R.

You may want to install this program using a 64 bit system.

This will allow you to later run the program using a 64 bit system.

Use the same file path you created in step 3 and indicated in step 4 above for your R libraries

No Windows binary version is available for CustomCDF at this time, so it must be installed on your Linux account.

**Information necessary to create masked U133 Plus2 gene expression data (Steps 7-11)**

**7. Change to the directory in which you have your .cel files stored and then read in your .cel files with the affy package.**

Type R to start an R session:

$ R

> library (affy)

This loads the affy library for handling gene expression data from Affymetrix microarrays

> library (CustomCDF)

This loads the R package for modifying the cdf environment appropriately

> library (hgu133plus2cdf)

This loads the cdf environment itself

To check if you have the appropriate .cel files in the current directory type:

> celFiles <- list.files(pattern="[.](c|C)(e|E)(l|L)$")

> celFiles

You should now see a list of the .cel files in your current directory.

> the_data <- ReadAffy()

The default settings for ReadAffy will read in all the cel files in the current directory. If you have many .cel files then your 64 bit Linux system is the best way to do this.

See the documentation for ReadAffy to learn more.

**8. Obtain the hgu133plus2 chimpanzee/human cross-species mask file provided as Supplementary File 2.**

Additional_File_7 is the master mask file that is included as an Additional File on the *BMC Bioinformatics* web site. Make sure this file is placed in the current directory.

If you are using any other Affymetrix platform you will have to create a similar type of file to progress to the next step.

> load ("Additional_File_7")

**9. Use the mask file to modify the environment and remove inappropriate probes**.

> removeprobe (the_data, pbMatrix=the_master_mask_file,minpbstsize=2)

The removeprobe command applies the mask to the affybatch object

The removeprobe command is part of the CustomCDF library

The minpbstsize refers to the minimum number of probes remaining in a probe set after masking in order for a gene expression score to be calculated

Type:

> ? removeprobe

The ? will display the documentation for the command if the library is currently loaded.

the_data is now a masked version of the data

**10. Check the work you have done so far.**

type "the_data" to view a description of the data.

You will see that there are now fewer probe sets remaining in the data if entire probe sets have been removed.

> the_data

AffyBatch object

size of arrays=1164x1164 features (21 kb)

cdf=HG-U133_Plus_2 (49956 affyids)

number of samples=55

number of genes=49956

annotation=hgu133plus2

notes=

> rm ("HG-U133_Plus_2")

Typing the above command will remove the mask from your data by removing the modified environment.

> the_data

AffyBatch object

size of arrays=1164x1164 features (21 kb)

cdf=HG-U133_Plus_2 (54675 affyids)

number of samples=55

number of genes=54675

annotation=hgu133plus2

notes=

Now apply a different version of the mask in which the minimum probe size is different.

> removeprobe (the_data, pbMatrix=the_master_mask_file, minpbstsize=5)

> the_data

AffyBatch object

size of arrays=1164x1164 features (21 kb)

cdf=HG-U133_Plus_2 (45402 affyids)

number of samples=55

number of genes=45402

annotation=hgu133plus2

notes=

**11. Continue with Bioconductor tools or export data for analysis in other software packages.**

The resulting masked AffyBatch objects can be used in many Bioconductor based analyses including normalization procedures such as rma and then with the limma package for differential expression analysis.

Or you could export your expression information as a text file at this time.

For example:

> normalized_data <- rma (the_data)

Once the data is normalized it is no longer tied to the cdf environment.

> write.exprs (normalized_data, file= "my_expression_text_file.txt")

You can export your normalized expression results in a text file and analyze the expression data using other computational tools.
